# Oncogenic BRAF(V600E) Induces Clastogenesis and UVB Hypersensitivity

**DOI:** 10.3390/cancers7020825

**Published:** 2015-06-17

**Authors:** Dennis A. Simpson, Nathalay Lemonie, David S. Morgan, Shobhan Gaddameedhi, William K. Kaufmann

**Affiliations:** 1 Department of Pathology & Laboratory Medicine, University of North Carolina at Chapel Hill, CB7295, Chapel Hill, NC 27599, USA; E-Mails: nlemoine@live.unc.edu (N.L.); dscardamone@gmail.com (D.S.M.); wkarlk@med.unc.edu (W.K.K.); 2 Lineberger Comprehensive Cancer Center, University of North Carolina at Chapel Hill, CB7295, Chapel Hill, NC 27599, USA; 3 Department of Experimental and Systems Pharmacology, College of Pharmacy, Washington State University, Spokane, WA 99210, USA; E-Mail: shobhan.gaddameedhi@wsu.edu; 4 Center for Environmental Health and Susceptibility, University of North Carolina at Chapel Hill, CB7295, Chapel Hill, NC 27599, USA

**Keywords:** BRAF(V600E), melanocytes, UVB, melanoma

## Abstract

The oncogenic BRAF(V600E) mutation is common in melanomas as well as moles. The roles that this mutation plays in the early events in the development of melanoma are poorly understood. This study demonstrates that expression of BRAF(V600E) is not only clastogenic, but synergizes for clastogenesis caused by exposure to ultraviolet radiation in the 300 to 320 nM (UVB) range. Expression of BRAF(V600E) was associated with induction of Chk1 pS280 and a reduction in chromatin remodeling factors BRG1 and BAF180. These alterations in the Chk1 signaling pathway and SWI/SNF chromatin remodeling pathway may contribute to the clastogenesis and UVB sensitivity. These results emphasize the importance of preventing sunburns in children with developing moles.

## 1. Introduction

The oncogenic BRAF(V600E) mutation is a common mutation in many cancers and precancerous lesions making it an important diagnostic marker and treatment target [1,2,3]. Of all cancers, the BRAF(V600E) mutation is most prevalent in melanoma, being observed in about 50% of melanomas and a similarly large fraction of melanocytic nevi [4,5]. Because the oncogenic BRAF(V600E) mutation is observed in precancerous nevi it is thought to be a very early mutation in the development of melanoma. With the epidemiological link between oncogenic BRAF(V600E) and melanoma being well established, the contribution of BRAF(V600E) to melanoma development is under intense investigation. *In vitro* expression of BRAF(V600E) in normal human cells, including melanocytes, results in a very rapid oncogene-induced growth arrest phenotype that is independent of p53 [6]. The effect of oncogenic BRAF(V600E) expression in nevi appears to be more complex, with a recent report suggesting that nevi may in fact not be as senescent as previously believed [7]. Slow replication of melanocytes in nevi may be necessary to fix into the genome mutations that are needed for the development of melanoma.

The Loeb mutator theory of human carcinogenesis holds that the background rate of mutation in human cells is too low to generate the 6–8 independent mutations that are needed to produce a cancer, so an early event in malignant transformation is a mutation (or epigenetic alteration) that increases the rate of mutation [8,9]. Previous reports have suggested that expression of BRAF(V600E) in cells may result in DNA damage as measured by micronuclei formation or by comet assay [10,11]. These studies both used proxies as a measure of the DNA damage present in the cell and neither suggested a mechanism for induction of the damage. A possible mechanism has been put forward that links BRAF(V600E), an oncogene, to BRCA1, a tumor suppressor [12]. In this model expression of BRAF(V600E) drives BRCA1 off the chromatin by down-regulation of BRIP1. This would make the cells phenotypically BRCA1 null as seen in reports where BRCA1 was excluded from the nucleus through expression of a dominant-negative BARD1 that blocks DNA damage-induced foci formation [13]. However expression of BRCA1 is growth-dependent and it is not clear if the observed reduction in BRCA1 was completely separate from the induction of oncogene-induced growth arrest. We report here the finding that expression of oncogenic BRAF(V600E) is directly clastogenic, potentially fulfilling the role of a Loeb mutator and that its expression sensitized cells to UVB-induced clastogenesis. We also present evidence for a model that expression of oncogenic BRAF(V600E) results in altered regulation of Chk1 and a reduction in the amount of BAF180, potentially explaining the clastogenesis and UVB sensitivity that is independent of oncogene-induced growth arrest.

## 2. Results and Discussion

### 2.1. Engineering and Validation of Cell Lines

**Figure 1:**
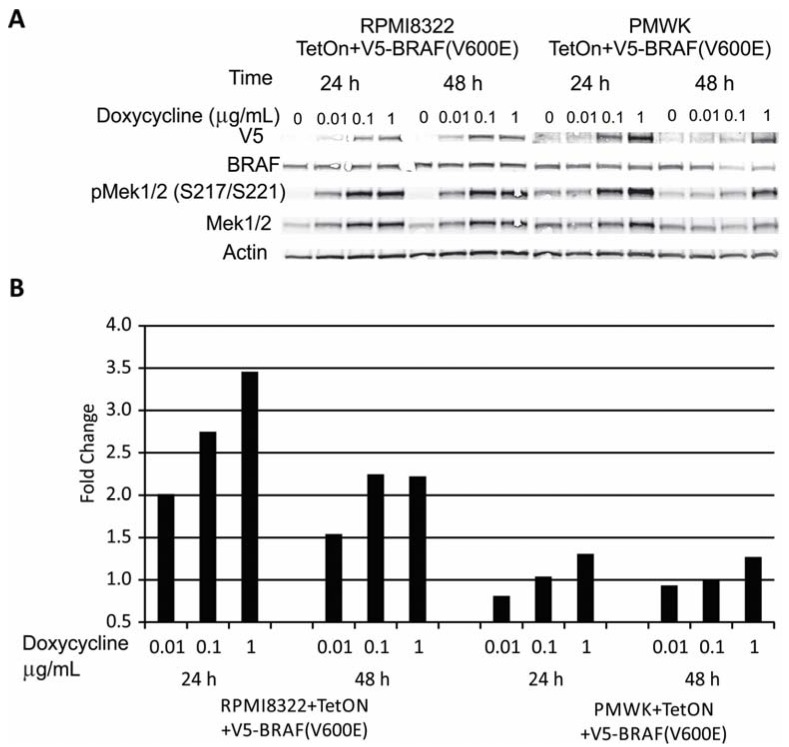
Induction of V5-tagged oncogenic BRAF(V600E) in RPMI8322 and PMWK cells. (**A**) Western Blot of whole cell lysates showing time and doxycycline concentration dependent induction of V5-BRAF(V600E). (**B**) Quantification of pMEK1/2 signal from panel A normalized to 0 µg/mL doxycycline control.

A melanoma cell line that is wild-type for *BRAF* and *NRAS*, and engineered to express oncogenic BRAF(V600E) has been described previously [14]. Using the same technique we engineered a second melanoma line that is wild-type for *BRAF* and *NRAS*. The DNA damage checkpoint responses have been described previously for both of these melanoma lines [15,16,17]. Neither has a functional G_1_ checkpoint response due to alterations in p53 signaling. Both have an intact G_2_ checkpoint response. Figure 1 shows that the expression of the V5-BRAF(V600E) can be regulated by the amount of doxycycline added to the medium in the two melanoma cell lines with the RPMI8322 + TetON + V5-BRAF(V600E) line exhibiting induction at a lower doxycycline concentration (0.01 μg/mL) than the PMWK + TetON + V5-BRAF(V600E) line (0.1 μg/mL). The western blot shown in Figure 1A demonstrated a clear increase in the amount of the V5 tag present as the amount of doxycycline was increased. The exogenous V5-BRAF was active as seen by the induction of phospho-MEK 1/2 and the correlation between activation of MEK1/2 and the amount of V5-BRAF present in the extract (Figure 1B). The RPMI8322 line not only exhibited a lower threshold for detection of V5-BRAF(V600E) but also exhibited a much greater fold induction of phospho-MEK1/2 than seen in the PMWK line.

**Figure 2:**
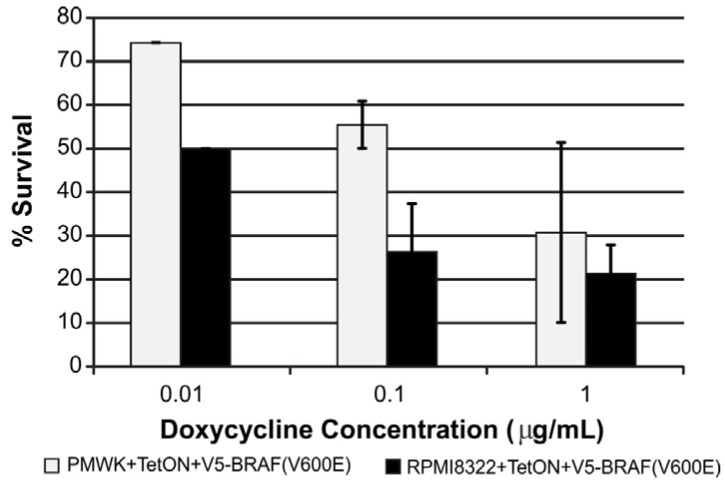
Oncogenic BRAF induced reduction in clonogenic expansion is dose dependent in all cell lines. Colony assay set up as described in the Experimental section. Each bar represents three to four independent determinations.

Since expression of oncogenic BRAF causes oncogene-induced growth arrest we tested whether this happened in our cell lines. As shown in Figure 2, expression of V5-BRAF(V600E) blocked clonal expansion of the cell lines in a dose-dependent manner. In agreement with the different levels of expression of V5-BRAF(V600E) shown in Figure 1A, the RPMI8322 line reduced colony formation to a greater extent than the PMWK line at the lower doxycycline concentrations. These results confirm both the biochemistry and biology of the system.

### 2.2. Oncogenic BRAF Induced Chromosomal Aberrations

**Figure 3:**
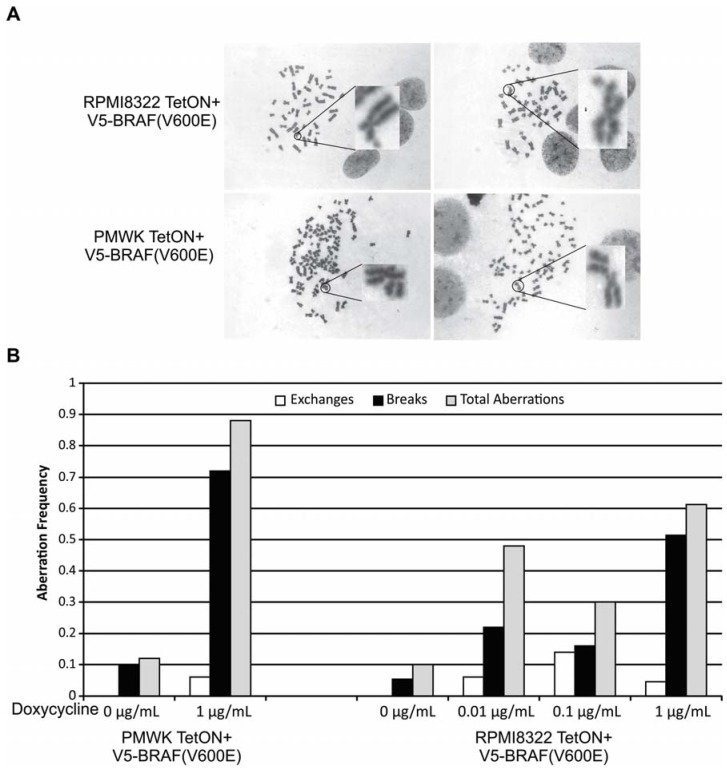
Oncogenic BRAF(V600E) Induced Clastogenesis. (**A**) Representative metaphase images of each cell line after 48 h incubation in doxycycline; (**B**) Aberrations per chromosome for each cell line. Data represents ≥50 metaphases for each treatment.

Previous reports have suggested that V5-BRAF(V600E) expression may result in chromosomal breaks and a DNA damage signal in cells as measured by comet assay, micronuclei formation, or induction of phosphorylated γH2aX [10,11]. We extended these observations by directly scoring metaphase preparations for damaged chromosomes. Expression of V5-BRAF(V600E) caused structural chromosomal aberrations in the RPMI8322 + TetON + V5-BRAF(V600E) and PMWK + TetON + V5-BRAF(V600E) cells after 48 h of induction. There was no detectable clastogenesis in either cell line after 24 h of oncogenic BRAF induction (not shown). Figure 3A shows representative examples of the types of damage observed. The aberrations observed in both cell lines were primarily chromatid breaks with a smaller number of exchange aberrations and a few dicentrics (Figure 3B). Even with this chromosomal damage and in contrast to published reports, we were unable to demonstrate induction of a DNA damage response as measured by phospho-ATM, phospho-Chk1 S345, or phospho-Chk2 T68 at 24 or 48 h induction with 1 μg/mL doxycycline (data not shown). This may be a result of the maximum aberration frequency being less than one aberration per metaphase which is equivalent to only a few cGy of ionizing radiation [18] or a combination of the different cell lines and different techniques employed (western blot in our studies, immunofluorescence in published studies).

Although both cell lines exhibit similar levels of chromosomal aberrations in the absence of V5-BRAF(V600E), the PMWK cell line exhibited a much higher break frequency than the RPMI cell line after induction. This may represent an underlying difference in chromatin architecture. The PMWK cell line was found to be polyploid with most metaphases having about 90 chromosomes while the RPMI cell line was near diploid. The total frequency of the aberrations in the RPMI line did not increase significantly with increasing expression of the oncogenic BRAF. The aberrations are unlikely to be due to an effect of the doxycycline since incubation of RPMI8322 + TetON cells, parents of the RPMI8322 + TetON + V5-BRAF(V600E) cells, with doxycycline did not increase aberrations (Table S1). This is the first direct evidence that oncogenic BRAF is clastogenic, making BRAF a potential mutator.

### 2.3. UVB Sensitivity of Cell Lines

**Figure 4:**
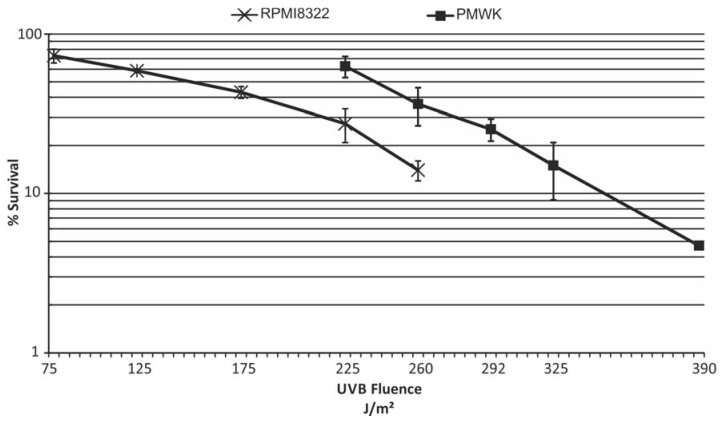
UVB cytotoxicity of RPMI8322 + TetON + V5-BRAF(V600E) and PMWK + TetON + V5-BRAF(V600E) cells. Cells were seeded at colony forming density and exposed to UVB 24 h later as described in the Experimental section. Expression of oncogenic BRAF was not induced in these cells. Each point represents three to four independent experiments.

Melanoma progression is linked to DNA damage caused by exposure to ultraviolet radiation [19]. Consistent with this is the observation that in a mouse model of melanoma, expression of oncogenic BRAF results in increased tumor burden following exposure to UVB [20]. Therefore it was of interest to determine if expression of V5-BRAF(V600E) sensitized cells to UV exposure. We wished to use a dose of UVB that did not kill all the cells (as dead cells do not play a role in melanomagenesis) but was in the realm of what would be expected for a person to receive from being outside during the summer in mid-latitudes. The sensitivity of the two melanoma cell lines to killing by UVB was determined as described in the Experimental section and is shown in Figure 4. From these data a D_0_ value, or dose to reduce the viable cell population to approximately 37%, was then calculated for each cell line according to published methods [21]. As previously published for UVC, the RPMI8322 line was found to be very sensitive to exposure to UVB, exhibiting a D_0_ of 191 J/m^2^, presumably due to defective nucleotide excision repair [22]. PMWK was found to have a D_0_ value of 254 J/m^2^. For subsequent experiments a fluence of UVB was chosen for each cell line that resulted in approximately equal killing.

### 2.4. UVB Induced Aberrations

**Figure 5:**
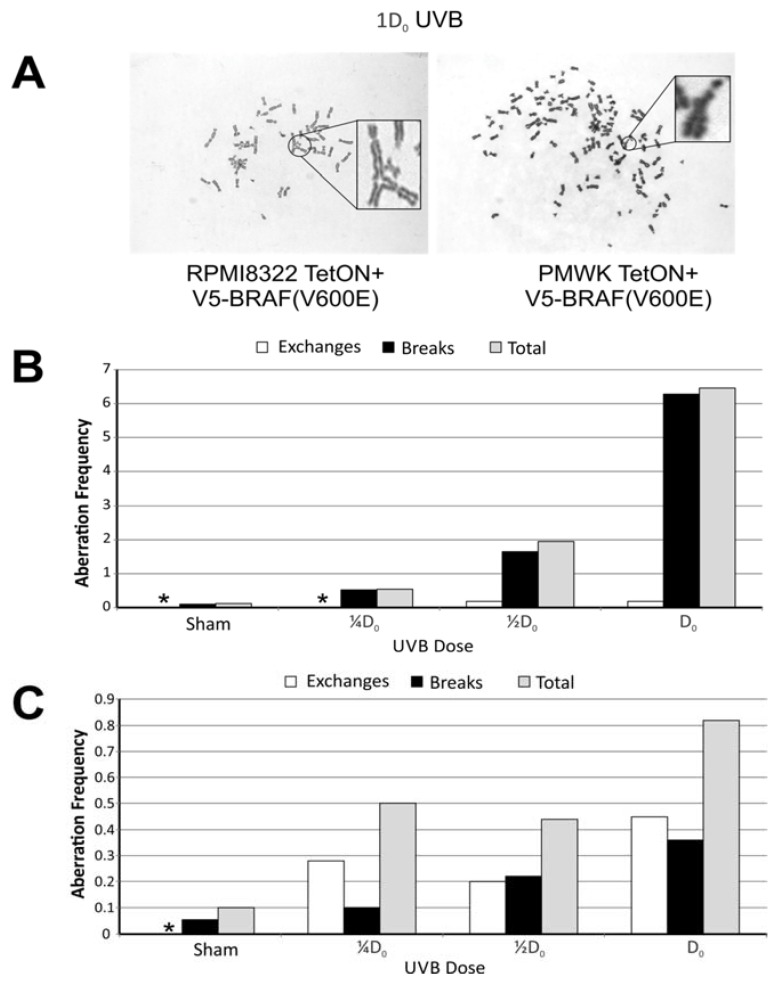
Chromosomal aberrations found 24 h after exposure to UVB. (*) No aberrations found. ≥50 metaphases scored for each condition. (**A**) Representative metaphase images showing aberrations; (**B**) Chart showing type of damage and frequency in PMWK + TetON + V5-BRAF(V600E) cells; (**C**) Chart showing type of damage and frequency in RPMI8322 + TetON + V5-BRAF(V600E) cells.

The melanoma cells were exposed to 0, ¼D_0_, ½D_0_, or D_0_ fluences of UVB 24 h after seeding the cells as described in the Experimental section. Metaphases were prepared 24 h after exposure, 48 h total culture time. The baseline clastogenesis resulting from UVB exposure in the absence of V5-BRAF(V600E) is shown in Figure 5. The background aberration frequencies in both cell lines were approximately equal, being 0.12 aberrations per metaphase and 0.1 aberrations per metaphase for the PMWK and RPMI8322 lines, respectively. UVB exposure resulted in an increase in the frequency of chromatid breaks and exchange aberrations in both cell lines that was dose dependent (Figure 5B,C). Overall the PMWK line exhibited a higher aberration frequency than the RPMI8322 line while the RPMI8322 line exhibited more exchange aberrations. The observation of the increased aberration frequency in the PMWK cell line was somewhat surprising given that this line is more resistant to UVB killing than the RPMI8322 line (Figure 4) and has measurable nucleotide excision repair capacity [22].

### 2.5. Oncogenic BRAF Sensitizes Cells to UVB Clastogenesis

**Figure 6:**
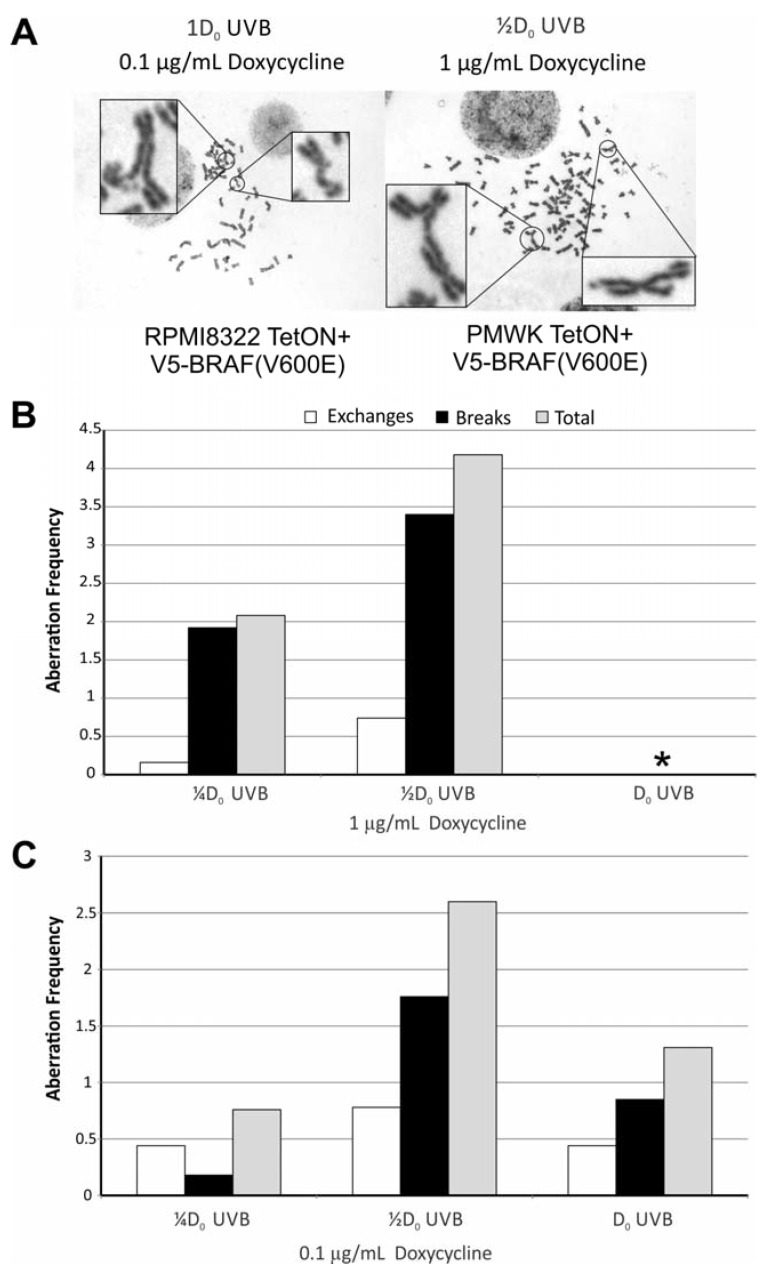
Expression of V5-BRAF(V600E) for 24 h followed by exposure to UVB results in enhanced clastogenesis. ≥50 metaphases scored for each condition. (**A**) Representative metaphase images showing aberrations. (**B**) PMWK + TetON + V5-BRAF(V600E) cells. (*) Metaphases were too damaged to score. >7 aberrations per chromosome. (**C**) Chart showing type of damage and frequency in RPMI8322 + TetON + V5-BRAF(V600E) cells.

To determine if expression of oncogenic BRAF had an effect on UVB clastogenesis, cells cultured in an amount of doxycycline (1 μg/mL PMWK and 0.1 μg/mL RPMI8322; Figure 1) that results in equal induction of V5-BRAF(V600E) and exposed to UVB as before were scored for chromosomal aberrations 24 h after UVB exposure. Representative metaphases are shown in Figure 6A for both cell lines. Like UVB alone, the PMWK (Figure 6B) line exhibited a break frequency much higher than the RPMI8322 line (Figure 6C). PMWK cells expressing oncogenic BRAF and exposed to a D_0_ of UVB could not be accurately scored. This dose of UVB in the presence of oncogenic BRAF resulted in very few scorable metaphases with every chromosome in those few metaphases containing multiple aberrations. At ¼D_0_ and ½D_0_ both lines exhibited a similar pattern of damage with the PMWK line now also showing exchange aberrations.

The fold-induction of chromosomal aberrations for the different treatments is shown in Table 1. The first three columns show the effect of UVB exposure at ¼D_0_ and ½D_0_ as well as the effect of oncogenic BRAF induction (Dox) on the PMWK and RPMI8322 cell lines. At the lowest fluence of UVB both cell lines exhibited a similar induction of aberrations. Combining the ¼D_0_ of UVB with oncogenic BRAF expression, the resulting induction of aberrations appears to be additive for both cell lines. This is seen in Table 1 where the frequency of aberrations induced by oncogenic BRAF alone was subtracted from the frequency of aberrations induced when oncogenic BRAF was combined with UVB exposure (columns 4 and 5). However, increasing the UVB dose to ½D_0_ in cells expressing oncogenic BRAF resulted in a fold-increase in the aberrations that was 3.6 and 7.6 times greater than that induced by doxycycline alone for the PMWK and RPMI8322 cells respectively (Table 1 column 5). At this dose of UVB, combining the two treatments resulted in a synergistic response. This increased sensitivity to ultraviolet light is likely the underlying reason the number of nevi a person carries coupled with sun exposure is the greatest risk factor for melanoma. This finding also fulfills the requirements for oncogenic BRAF to impart a mutator phenotype to cells harboring the mutation, as postulated by Loeb and colleagues [8,9].

**Table 1:** Effect of Oncogenic BRAF on UVB Induced Aberrations.

	Treatment/Sham			(Freq-Dox Freq)/Sham	
	¼D_0_	½D_0_	* Dox	¼D_0_ + * Dox	½D_0_ + * Dox
^§^ PMWK	4.5	16	7.3	10	28
^†^ RPMI8322	5.0	4.4	3.0	4.6	23

The first three columns show the induction of aberrations caused by UVB or oncogenic BRAF alone. The last two columns show the induction of aberrations attributable to oncogenic BRAF in cells exposed to both UVB and dox. (§) PMWK + TetON + V5-BRAF(V600E) cells. (†) RPMI8322 + TetON + V5-BRAF(V600E) cells. (*) Doxycycline. 1 µg/mL for PMWK cells; 0.1 µg/mL for RPMI8322 cells.

### 2.6. Mechanisms of BRAF(V600E) Induced Sensitivity

The mechanism responsible for the oncogenic BRAF-induced aberrations and the synergy with UVB is not obvious. To determine if the sensitivity to UV observed in the oncogenic BRAF-expressing cells was due to alterations in the repair capacity of the cells we measured nucleotide excision repair (NER) in the V5-BRAF(V600E) expressing RPMI and PMWK cells exposed to a D_0_ dose of UVB. Cells were assayed for repair of CPD’s and 6-4PP as previously described [22]. Induction of oncogenic BRAF did not alter repair of UVB-induced DNA damage in these cells (data not shown).

#### 2.6.1. Alteration of BRCA1 Function

**Figure 7:**
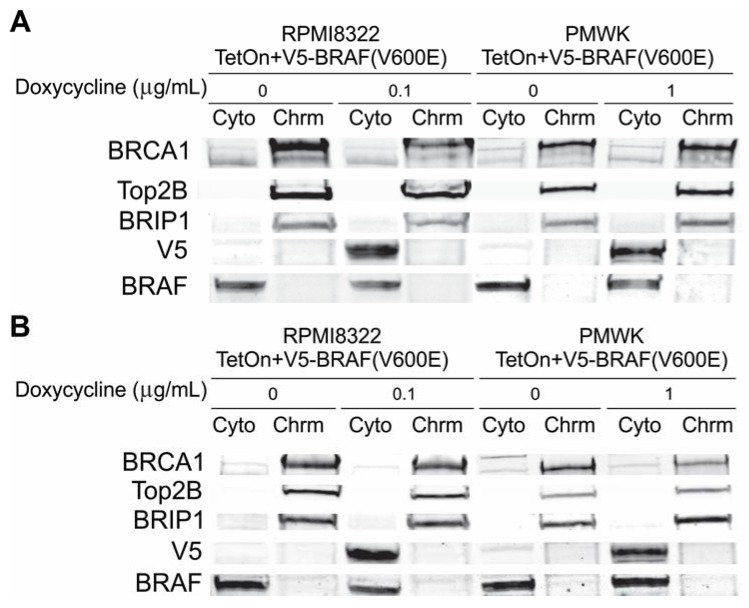
Western blot cells grown for (**A**) 24 h in doxycycline or (**B**) 48 h in doxycycline. Loading normalized to cell number. Cyto cytoplasm; Chrm chromatin fraction. Fractionation described in the Experimental section.

The exchange aberrations and breaks observed after induction of V5-BRAF(V600E) and the increased sensitivity to UVB suggested that misregulation of BRCA1 might be a possible cause. BRCA1 is required for efficient repair of UV damage [23] as well as regulation of homologous recombination [24]. Indeed a publication suggested that oncogenic BRAF could reduce the amount of BRCA1 associated with chromatin by down-regulation of BRIP1 [12]. We measured the amount of cytoplasmic and chromatin associated BRCA1 and BRIP1 in cells 24 and 48 h after induction of the V5-BRAF(V600E). In three independent experiments using RPMI8322 + TetON + V5-BRAF(V600E) and PMWK + TetON + V5-BRAF(V600E) cell lines, we found that that amount of BRCA1 in the cells after V5-BRAF(V600E) induction was variable and did decrease with time in doxycycline. Figure 7 shows a representative western Blot from these experiments. To explore the possibility that this variability was due to the cell type, we constructed a normal human fibroblast line to express V5-BRAF(V600E) as described in the Experimental section. This line induces V5-BRAF(V600E) similar to that of PMWK + TetON + V5-BRAF(V600E) and this induction causes oncogene-induced growth arrest as seen by an inhibition in colony formation (Figure S1). The location and amount of BRCA1 and BRIP1 in the fibroblast line was consistent with that observed in Figure 7 (Figure S2). These results suggested the possibility that the amount of BRCA1 might be varying as a result of how well the cells were growing.

**Figure 8:**
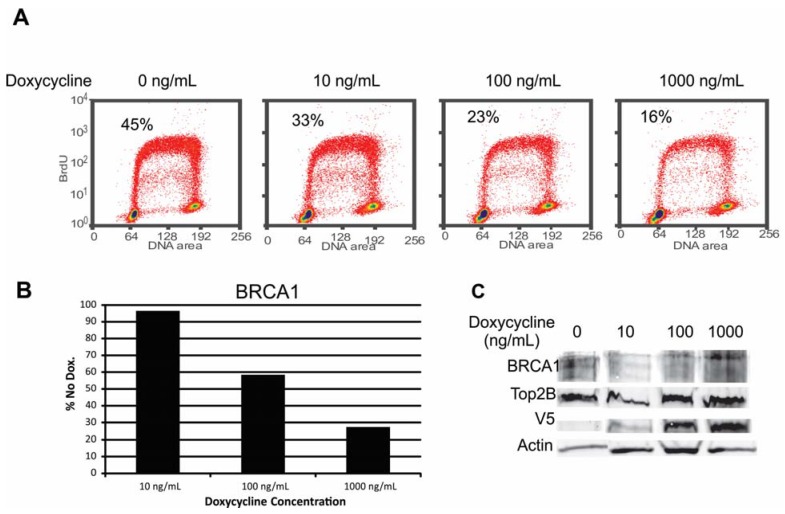
Correlation of the amount of BRCA1 present in RPMI8322 + TetON + V5-BRAF(V600E) cells with S-phase fraction of the cell population. (**A**) FLOW cytometry profiles of cells incubated with increasing amounts of doxycycline. (**B**) Amount of BRCA1 present as a percentage of the no doxycycline control. (**C**) Western Blot used to generate chart in panel B.

BRCA1 is known to exhibit cell cycle dependent expression [25,26]. To test whether proliferation affected expression of BRCA1 RPMI8322 + TetON + V5-BRAF(V600E) cells were grown with increasing amounts of doxycycline and labeled with BrdU to determine the S-phase content prior to harvest. Figure 8 shows the total amount of BRCA1 in RPMI8322 + TetON + V5-BRAF(V600E) cells, normalized to topoisomerase IIβ, a chromatin marker, went down as the amount of V5-BRAF(V600E) went up. However, the highest induction of V5-BRAF(V600E) resulted in an 84% reduction in S-phase and a 73% reduction in the amount of BRCA1 in the cells. Based on these results we could not rule out oncogene-induced growth arrest as the cause of the reduction in BRCA1 expression.

#### 2.6.2. Alteration of Chk1 Signaling

**Figure 9:**
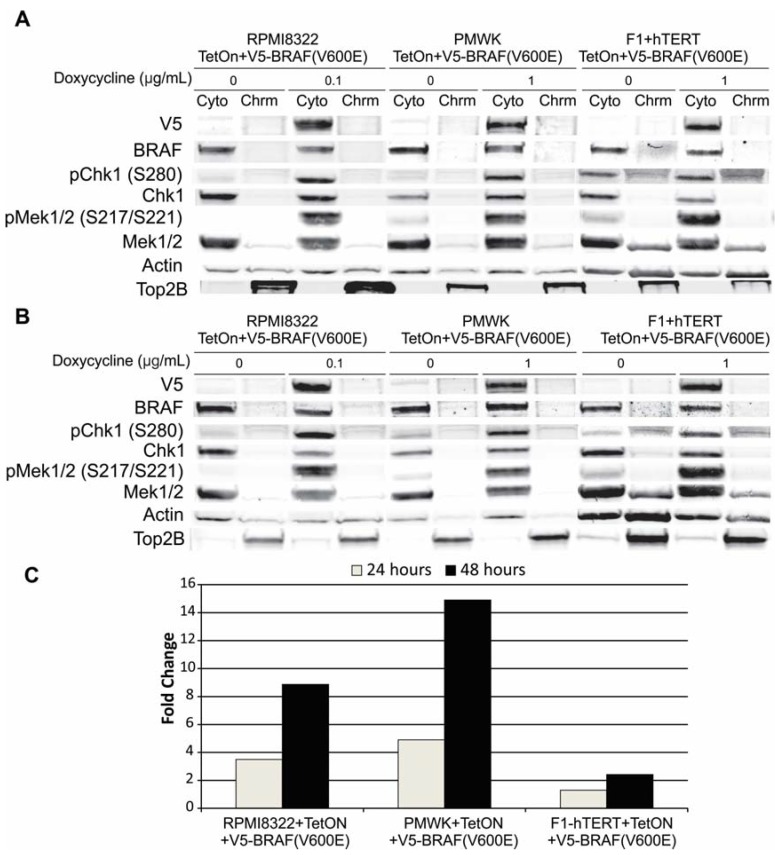
Induction of Chk1 pS280 by V5-BRAF(V600E). Cells were incubated in the amount of doxycycline shown for 24 h (panel **A**), or 48 h (panel **B**). Panel **C** is a chart of the fold-induction of Chk1 pS280 normalized to actin and total Chk1.

While it did not appear likely that the amount or location of BRCA1, or alterations in NER, were responsible for the oncogenic BRAF-induced clastogenesis or UVB sensitivity, another possibility was our previously published observation that expression of oncogenic BRAF attenuates the DNA damage-induced G2 checkpoint response [14]. Chk1 is a central protein in the G2 checkpoint response. Any alterations in the regulation of Chk1 could impact this checkpoint. Consistent with the possibility of Chk1 misregulation was the observation with all three cell lines that induction of V5-BRAF(V600E) always resulted in a substantial increase in the amount of pChk1 (S280). Figure 9 is a composite western blot of all three cell lines grown with or without doxycycline for 24 or 48 h. As shown in Figure 9C, all three cell lines showed an increase in the amount of pChk1 (S280) at both 24 and 48 h. The fold increase in the F1-hTERT + TetON + V5-BRAF(V600E) cells was always slightly less than that seen in the two melanoma lines. The apparent cause of this is the fibroblasts have a slightly higher amount of pChk1 (S280) in the uninduced cells.

Chk1 S280 is phosphorylated by AKT1 [27] and p90RSK1 [28]. Induction of Chk1 pS280 through active AKT1 by inhibition or depletion of PTEN has been shown to result in a reduction in pChk1 pS345 kinase activity one hour after exposure to UVC and a reduced amount of pChk1 pS354 24 h after exposure [27,29]. Consistent with this was the observation that Chk1 phosphorylated at S280 was less likely to form complexes with other proteins [30]. The S280 phosphorylation of Chk1 in the oncogenic BRAF-expressing cells is unlikely to proceed through AKT1 as oncogenic BRAF inhibits AKT1 activity in melanoma cells and probably any cell through inhibition of RICTOR [31]. However, at the same time oncogenic BRAF activates p90RSK1 through the phosphorylation of p90RSK1 by ERK [32] which would then lead to phosphorylation of Chk1 on S280. Similar to the finding that AKT1 induced Chk1 pS280 results in an attenuation of Chk1 kinase activity, another study using melanoma cells found that inhibition of RSK1 or MEK results in a reduction in the amount of Chk1 pS280 and an increase in the amount of Chk1 kinase activity [33].

#### 2.6.3. Alteration of SWI/SNF Chromatin Remodeling Complex

**Figure 10:**
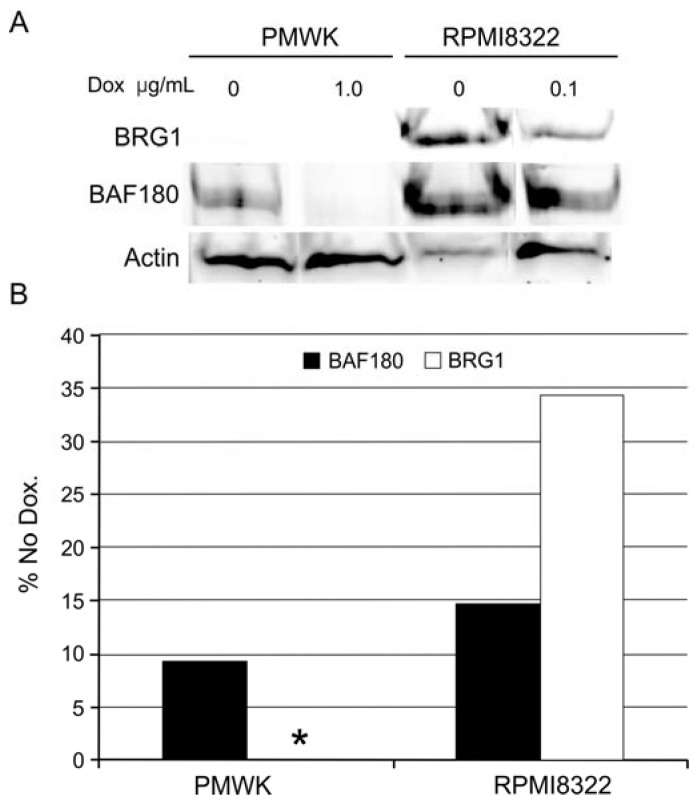
Reduction in BRG1 and BAF180 in cells expressing oncogenic BRAF. PMWK: PMWK + TetON + V5-BRAF(V600E) cells; RPMI8322: RPMI8322 + TetON + V5-BRAF(V600E) cells. (**A**) Western blot of whole cell lysates of cells grown for 48 h in doxycycline. (*) PMWK does not express BRG1 protein. (**B**) Quantification of western blot, with pixel density normalized to actin.

A final possibility for explaining the clastogenesis induced by the oncogenic BRAF and the synergy with UVB exposure is alteration of the SWI/SNF chromatin remodeling complex. As shown in Figure 3 and Figure 5 and Figure 6 the PMWK cell line exhibited higher frequencies of chromosomal aberrations from oncogenic BRAF or UVB alone and when combined. A possible explanation for this observation is that the PMWK cells do not express BRG1 (Figure S3). Loss of BRG1 has been shown to increase the frequency of DNA damage after exposure to UVC [34]. When all the cells used in this study were examined for BRG1 and BAF180 expression it was found that expression of oncogenic BRAF reduced the expression of BRG1 in the two cell lines that expressed it and reduced the expression of BAF180 in all three cell lines (Figure 10).

The synergistic induction of chromosomal aberrations by UVB in cells expressing oncogenic BRAF suggests that developing moles are sensitive targets for UVB-induced chromosomal damage. As melanocytic nevi commonly develop during childhood and most nevi express oncogenic BRAF, strategies for prevention of melanoma must emphasize protecting children against damaging sunburns. This study also suggests possible implications in other cancers such as colon cancer where the oncogenic BRAF mutation has been reported in 70% of the tumors exhibiting the highest level of microsatellite instability [35]. This may imply that the oncogenic BRAF may act synergistically in other cancers to ultimately increase the mutation frequency in these tumors.

## 3. Experimental Section

### 3.1. Molecular Biology

A V5-BRAF(V600E) expression vector was constructed by cloning a synthetic V5-BRAF(V600E) DNA cassette into the pRetro-X-Advanced (Clonetech, Mountain View, CA, USA) vector. Vector construction and sequence verification of the vector was done by Blue Heron Bio (Bothell, WA, USA).

### 3.2. Cell Culture and Viral Transduction

Melanoma cell lines RPMI8322 and PMWK were grown in DMEM high glucose (Life Technologies, Carlsbad, CA, USA or Cellgro, Manassas, VA, USA) supplemented with 10% Benchmark fetal calf serum (Gemini, West Sacramento, CA, USA), and 200 mM L-glutamine (Life Technologies) at 37 °C in a 5% CO_2_ atmosphere. Packaging of retroviral vectors and transduction of cells was done as previously described [36]. The cell lines were first transduced with the Retro-X-TetON vector (Clonetech). Following selection in 500 μg/mL G418 for two weeks the cells were then transduced with the V5-BRAF(V600E) expression vector. The V5-BRAF(V600E) vector was selected for two weeks in 150 μg/mL hygromycin B.

### 3.3. Cytotoxicity Assays

The ability of the oncogenic V5-BRAF(V600E) to reduce clonogenic survival was assessed as follows. Cells were trypsonized to achieve a single cell suspension. The viable cell count was determined by trypan blue exclusion and counting cells in a heamocytometer. Three hundred viable cells were seeded per 10 cm diameter dish in triplicate in 10 mL of media containing various amounts of doxycycline. The media was changed on day seven, maintaining the doxycycline. On day 14 the colonies were fixed and stained with a solution of 40% methanol and 0.05% crystal violet.

UVB cytotoxicity was determined by seeding 300 viable cells per dish as above. Twenty-four hours after seeding the media was removed and the cells exposed to various fluences of UVB with the lids on the dishes in an open cabinet to the output from two high intensity broad range UVB lamps (FS20T12/UVB, National Biological Corp., Cleveland, OH, USA) at a fluence of 3.13 J/s/m^2^. By keeping the lids on the dishes all wavelengths less than about 300 nM were filtered out resulting in a UVB profile more similar to that found in sunlight [37]. The UVB source was calibrated with a UVX Radiometer (Ultra-Violet Products, Inc., Upland, CA, USA) using an UVX-31 sensor. The cells were grown for 14 days after exposure and then stained as described above. Calculations to determine the D_0_ value were done as previously published [21].

### 3.4. Western Blotting

Whole cell extracts were prepared by solubilizing cell pellets in urea buffer (8 M urea, 100 mM monobasic sodium phosphate, 10 mM tris pH 7.0) for 30 min on ice prior to protein quantification. Proteins were quantified using a Qubit^®^ (Life Technologies) according to manufactures protocol. Cells were fractionated into cytoplasmic and chromatin fractions by previously published methods [38]. Primary antibodies for western blots used in this study are listed in Table 2.

**Table 2:** Primary antibodies used in the study.

Antibody	Dilution	Source	Catalogue Number
BRCA1	1:1000	Milli-Pore	07-434
Top2β	1:1000	BD Transduction Labs	611493
BRIP1/BACH1	1:1000	Cell Signaling	4578
BRG1	1:1000	Bethyl	A300-813A
BRAF	1:1000	Cell Signaling	9434
V5	1:1000	Sigma-Aldrich	012M4796
Chk1	1:1000	Santa Cruz	SC-8408
pChk1 (S345)	1:1000	Cell Signaling	2348
pChk1 (S280)	1:1000	Epitomics	2643-1
Mek1/2	1:1000	Cell Signaling	4694
pMek1/2 (S217/S221)	1:1000	Cell Signaling	9154
Pan-Actin	1:5000	Novus Biologics	NB600-535

The primary antibodies were detected using fluorescently labeled anti-mouse and anti-rabbit secondary antibodies at a dilution of 1:10,000 and obtained from Li-Cor (Goat Anti-Mouse IRDye 680RD; Cat.#926-68070 and Goat Anti-Rabbit IRDye 800CW; Cat.#926-32211). The proteins were visualized by scanning the blots on a Li-Cor Odyssey^®^ (Lincoln, NE, USA scanner. Images were quantified using the Visual Studio 4.0 software package (Li-Cor) unless noted otherwise.

## 4. Conclusions

Expression of oncogenic BRAF(V600E) was clastogenic in two human melanoma cell lines. The melanoma cell lines provided an opportunity to study mitotic chromosome structure after induction of the oncogene but prior to cessation of cell division. Equivalent studies were done with a TERT-expressing, immortal human melanocyte line but no mitotic cells could be recovered two days after induction of BRAF(V600E) (results not shown). As BRAF(V600E) attenuated the DNA damage G2 checkpoint [14] it may be that the relaxation of this checkpoint permitted the analysis of mitotic chromosomes in addition to sensitizing cells to UVB-induced clastogenesis. Oncogenic BRAF(V600E) sensitized the melanoma cells to exposure to doses of UVB that would be expected to result in only mild erythema in most Caucasians [39]. The type of DNA damage observed in the BRAF(V600E) expressing cells exposed to UVB radiation was similar to that observed in cells lacking normal BRCA1 regulation [23]. This is consistent with a publication demonstrating that oncogenic RAS and BRAF both cause misregulation of BRCA1 through a reduction in BRIP1 [12]. However, we were unable to demonstrate that this was due to BRAF and instead conclude that the reduction in BRCA1 was a result of cells exiting the cell cycle (Figure 8).

We have previously reported that expression of oncogenic BRAF attenuates the DNA damage induced G2 checkpoint response [14]. Oncogenic BRAF induced phosphorylation of Chk1 at S280 (Figure 9). This form of Chk1 has been reported to be altered in its function or regulation [27,29,30]. Finally, in PMWK + TetON + V5-BRAF(V600E) cells, induction of oncogenic BRAF alone, exposure to UVB alone or induction of oncogenic BRAF combined with exposure to UVB resulted in more chromosomal aberrations than that observed in the RPMI8322 + TetON + V5-BRAF(V600E) cells. Both these lines induced Chk1 pS280 equally suggesting that the potential altered regulation of Chk1 was not the entire mechanism. Alterations in the SWI/SNF chromatin remodeling complex have been reported to sensitize cells to UV radiation and attenuate the DNA damage G2 checkpoint response [34,40]. PMWK + TetON + V5-BRAF(V600E) cells were found to not express BRG1. Unexpectedly RPMI8322 + TetON + V5-BRAF(V600E) cells were found to reduce the amount of BRG1 after induction of oncogenic BRAF (Figure 10). Both cell lines exhibited reduced levels of BAF180 following V5-BRAF(V600E) expression. This is the first report linking the MAP kinase pathway to the chromatin remodeling pathway. Expression of oncogenic BRAF in cells results in elevated levels of Chk1pS280 and reduced levels of BRG1 and BAF180 associated with attenuation of the G2 checkpoint response, clastogenesis, and UVB sensitivity. While the cells used in this study were melanoma cells it is plausible that this phenomenon will occur in melanocytes contained within nevi, since these melanocytes primarily contain the oncogenic form of BRAF. Further studies will be required to establish the potential role of BRAF induced clastogenesis and BRAF induced UVB sensitivity to the progression of melanoma from nevi. However these data present an intriguing possibility for explaining the link between the risk associated with increased nevi and the number of sunburns.

## Supplementary Files

Supplementary File 1
