# Supplementary material for: Oncogenic BRAF(V600E) Induces Clastogenesis and UVB Hypersensitivity
Source: Cancers (Basel). 2015 Jun 17;7(2):1072–90. doi: 10.3390/cancers7020825 (PMC4491700; doi:10.3390/cancers7020825)
Supplement: Supplementary File 1 [file cancers-07-00825-s001.pdf]

Supplementary Materials

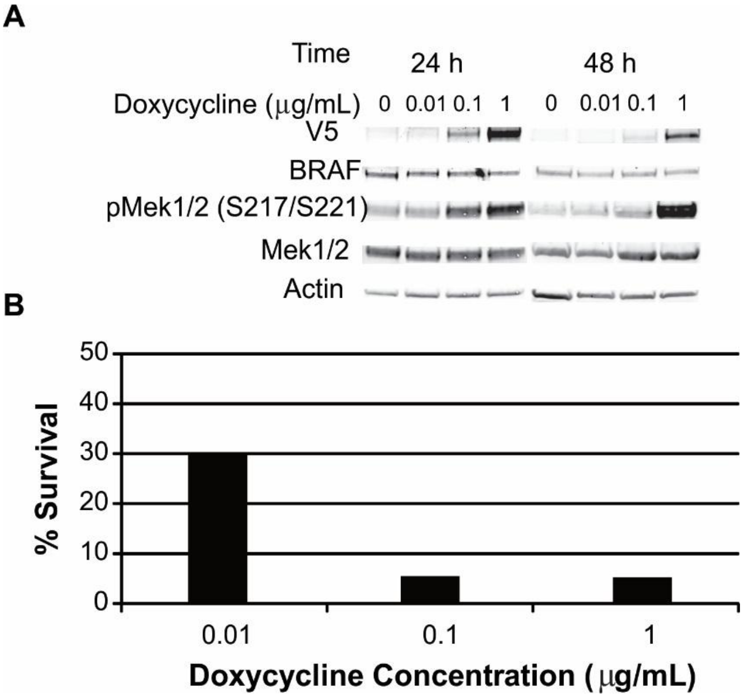

Figure S1. F1-hTERT + TetON + V5-BRAF(V600E) cells grown in the amount of doxycycline shown. (**A**) Western Blot is whole cell lysates after 24 or 48 h in doxycycline. (**B**) Inactivation of colony formation by induction of oncogenic BRAF.

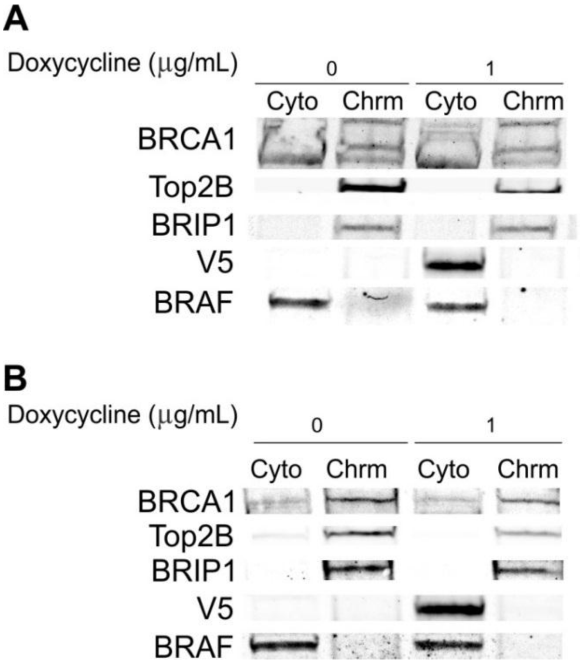

Figure S2. F1-hTERT + TetON + V5-BRAF(V600E) cells grown for (**A**) 24 h or (**B**) 48 h in doxycycline. Cyto cytoplasm; Chrm chromatin fraction. Cells fractionated according to methods. Each lane of the Western Blot is loaded with an equal cell number. Blot shows a modest reduction in the amount of chromatin associated BRCA1 after 48 h.

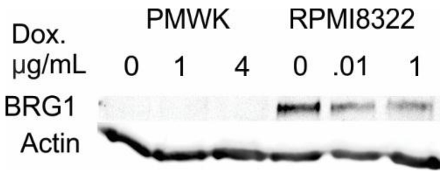

Figure S3. Western Blot of whole cell lysates. PMWK: PMWK + TetON + V5-BRAF(V600E) cells; RPMI8322: RPMI8322 + TetON + V5-BRAF(V600E) cells. The PMWK + TetON + V5-BRAF(V600E) cells do not express any BRG1 protein and oncogenic BRAF expression reduces the amount of BRG1 in the RPMI8322 cell line.

Table S1. Aberration frequencies of the parental RPMI8322 + TetON cell line ±1 µg/mL doxycycline, ±UVB (1D_0). Cells were treated as described in the methods. As compared to the RPMI8322 line without doxycycline in Figure 5, doxycycline had no effect on the induction or frequency of aberrations.

	**Frequency**		
**Treatment**	**Breaks**	**Exchanges**	**Total Aberrations**
Sham	0.03	0.00	0.04
Sham + UVB	1.1	0.94	2.0
1 µg/mL Doxycycline	0.04	0.00	0.06
1 µg/mL Doxycycline + UVB	0.66	0.51	1.2

© 2015 by the authors; licensee MDPI, Basel, Switzerland. This article is an open access article distributed under the terms and conditions of the Creative Commons Attribution license (http://creativecommons.org/licenses/by/4.0/).
